# Peripheral antibody concentrations are associated with highly differentiated T cells and inflammatory processes in the human bone marrow

**DOI:** 10.1186/s12979-019-0161-z

**Published:** 2019-08-22

**Authors:** Erin Naismith, Luca Pangrazzi, Marco Grasse, Michael Keller, Carina Miggitsch, Birgit Weinberger, Klemens Trieb, Beatrix Grubeck-Loebenstein

**Affiliations:** 1 Institute for Biomedical Aging Research, University of Innsbruck, Rennweg 10, A-6020 Innsbruck, Austria; 2 Department of Orthopedic Surgery, Hospital Wels-Grieskirchen, Grieskirchnerstrasse 42, Wels, Austria

**Keywords:** Bone marrow, Aging, Inflammation, Pro-inflammatory, Immunosenescence, Senescence, Exhaustion, Antibodies, B cells

## Abstract

**Background:**

Antigen-experienced immune cells migrate back to the bone marrow (BM), where they are maintained in BM survival niches for an extended period. The composition of T cell subpopulations in the BM changes with age, leading to an accumulation of highly differentiated T cells and a loss of naïve T cells. While innate immune cells are also affected by age, little is known about interactions between different adaptive immune cell populations maintained in the BM. In this study, the phenotype and function of innate and adaptive immune cells isolated from human BM and peripheral blood (PB) was analysed in detail using flow cytometry, to determine if the accumulation of highly differentiated T and B cells, supported by the BM niches, limits the maintenance of other immune cells, or affects their functions such as providing protective antibody concentrations.

**Results:**

Total T cells increase in the BM with age, as do highly differentiated CD8^+^ T cells which no longer express the co-stimulatory molecule CD28, while natural killer T (NKT) cells, monocytes, B cells, and naïve CD8^+^ T cells all decrease in the BM with age. A negative correlation of total T cells with B cells was observed in the BM. The percentage of B cells in the BM negatively correlated with highly differentiated CD8^+^CD28^−^ T cells, replicative-senescent CD8^+^CD57^+^ T cells, as well as the CD8^+^CD28^−^CD57^+^ population. Similar correlations were seen between B cells and the frequency of highly differentiated T cells producing pro-inflammatory molecules in the BM. Interestingly, plasma concentrations of diphtheria-specific antibodies negatively correlated with highly differentiated CD8^+^CD57^+^ T cells as well as with exhausted central memory CD8^+^ and CD4^+^ T cells in the BM. A negative impact on diphtheria-specific antibodies was also observed for CD8^+^ T cells expressing senescence associated genes such as the cell cycle regulator p21 (CDKN1A), KLRG-1, and elevated levels of reactive oxygen species (ROS).

**Conclusion:**

Our data suggest that the accumulation and maintenance of highly differentiated, senescent, and exhausted T cells in the BM, particularly in old age, may interfere with the survival of other cell populations resident in the BM such as monocytes and B cells, leading to reduced peripheral diphtheria antibody concentrations as a result. These findings further highlight the importance of the BM in the long-term maintenance of immunological memory.

**Electronic supplementary material:**

The online version of this article (10.1186/s12979-019-0161-z) contains supplementary material, which is available to authorized users.

## Introduction

Activated B and T cells differentiate into memory and effector cells, and can either proceed to the area of infection, circulate through the blood and lymph, or return to the peripheral lymphoid organs [1]. In addition, many immune cells migrate back to the bone marrow (BM), where they can remain in different activation states for an extended period [2]. Different states of differentiation and activation can be characterized by marker proteins that these immune cells secrete or express on their surface [3]. The BM is well known for haematopoiesis and its function as a primary lymphoid organ, however its role in the long-term maintenance of antigen-experienced immune cells is less well understood. The BM is involved in the regulation, function, and survival of plasma cells, as well as memory B and T cells [4]. While the number of CD4^+^ and CD8^+^ T cells in the BM is maintained during aging, the composition of subpopulations changes, showing an increase in highly-differentiated effector memory cells and a decrease in naïve cells [5]. CD28 is a co-stimulatory molecule found on the surface of T cells which provides the secondary signal upon T cell activation [6]. Cells that lose CD28 are generally antigen-experienced, highly differentiated, pro-inflammatory, and are preferentially maintained in the BM by IL-15 [7–9]. CD8^+^CD28^−^ T cells are known to increase in the BM with aging, and under some conditions they acquire the expression of CD57, a marker for replicative senescence and terminal differentiation [10].

As the space within the BM is restricted, and different populations share the same survival factors, a question of interest is whether the accumulation of some immune cells may interfere with the maintenance of other subsets. In particular, highly differentiated T cells in the BM may interfere with the maintenance of other populations such as monocytes and B cells, and alter the BM environment with the production of pro-inflammatory molecules. As IL-15 expression increases with aging [7, 11], and it is known to be important for the survival of CD8^+^CD28^−^ T cells, we hypothesized that, in old age, BM niches may recruit high frequency of highly differentiated CD8^+^ T cells, limiting the space for other cell types [7]. The B cell compartment in the BM is also affected by age and plasma cell numbers decrease [12]. Distinct niches provided by cytokine-producing stroma cells have also been described for murine memory CD4^+^ T cells [13].

Cellular senescence and exhaustion represents typical hallmarks of aging [14]. Senescent cells secrete soluble and insoluble factors such as interleukins, chemokines, fibronectin, and collagens [15], which modulate signalling pathways associated with inflammation and malignancies, in addition to directly secreting pro-inflammatory cytokines inducing low-grade chronic inflammation, which when present in the BM may affect its ability to harbor long-living immune cells [7, 16]. Cellular senescence is a process by which the emergence of transformed cells is prevented via permanent cell cycle arrest, during which the cells remain metabolically active [17]. p21 is a central regulator of cell cycle progression and it is a major target of the p53 pathway, which is activated by DNA damage or other stresses [18]. p21 promotes cell cycle inhibition, protect cells from apoptosis, and can thereby be used as a reliable marker for senescence [19]. In addition to p21, killer cell lectin-like receptor subfamily G, member 1 (KLRG-1) represents a marker for T cell senescence. KLRG-1 expression on CD8^+^ T cells indicates a subpopulation which is unable to undergo further cell division and is therefore terminally differentiated or replicatively senescent [20]. KLRG-1 is negatively regulated by programmed cell death protein 1 (PD-1) [21]. PD-1 is a characteristic marker for exhaustion [22], and was initially recognized for its ability to induce apoptosis [23], however it should not be regarded as a definitive marker for exhausted cells in general [24]. PD-1 is an inhibitory receptor which is expressed on the surface of activated T cells, and is maintained during chronic infection [25]. PD-1 has two ligands, PDL-1 and PDL-2, which upon binding activate inhibitory signals for cell cycle progression [21] and impair T cell receptor (TCR) signalling [23]. Positive correlations have been observed between PD-1 and the proliferation marker Ki67 on memory CD8^+^ and CD4^+^ T cells, and negative correlations between PD-1 density and Ki67 expression in central memory (CM) CD8^+^ T cells [24], therefore the presence of PD-1 on non-proliferating cells, such as CM cells, indicate that they are exhausted [23]. The expression of p21, KLRG-1, and PD-1 are all known to increase with age [16].

These antecedents have led to the hypothesis that the accumulation and maintenance of effector, senescent, and/or exhausted T cells in aged BM may disrupt or alter the immunological function of the BM. We consider the consequential displacement of other cell types, such as CD4^+^ T cells, B cells, and plasma cells, due to competition for stromal niches. Long-lived plasma cell survival is mediated by stromal cells in the BM [26]. Therefore we hypothesized that changes in the BM environment may lead to impaired antibody production. To assess this, the concentration of Diphtheria-specific antibody was measured in PB. As Diphtheria-specific antibodies are not maintained well with aging [27], we hypothesized that the competition for space, as well as the age-related changes taking place in the BM environment in old age, may directly contribute to decreased immune responses against Diphtheria observed in the elderly.

In the current study, we investigated whether competition for space between B and T cell subpopulations takes place in the BM. In addition, we assessed whether the accumulation of highly differentiated CD8^+^ T cells, which have been described to support inflammation and oxidative stress in the BM [11], may be negatively associated with the maintenance of long-lived plasma cells, affecting the production of diphtheria-specific antibody in the periphery as a result.

## Materials and methods

### Sample preparation

Samples were obtained from systemically healthy individuals who do not suffer from diseases known to affect the immune system. All samples were obtained from people undergoing elective surgeries because of osteoarthrosis. The donors comprised of 95 individuals aged between 39 and 87 (mean age: 67.45 ± 10.95, mean BMI: 27.9 ± 5.03, sex: 50 F, 46 M). The number of samples used in individual experiments are given in the figures and legends.

For the isolation of bone marrow mononuclear cells (BMMCs), a fragment of *substantia spongiosa osseum,* which would otherwise be discarded was collected during routine hip replacement surgery. The bone was further fragmented and treated with purified collagenase (CLSPA, Worthington Biochemical; 20 U/ml) in complete RPMI medium (RPMI 1640; Corning supplemented with 10% FCS, 100 U/ml penicillin, and 100 μg/ml streptomycin; Sigma) for 1 h at 37 °C. BMMCs were extracted using a filtered tube centrifugation step, and then purified using density gradient centrifugation (Lymphoprep®; Stemcell technologies). Heparinised blood from the same donors was collected, and peripheral blood mononuclear cells (PBMCs) were also purified by density gradient centrifugation.

### Flow cytometry

Immunofluorescence stainings were done using conjugated surface antibodies. BMMCs and PBMCs were incubated with flourochrome-labeled antibodies for 20 min at 4 °C. Cells were washed with PBS and measured using a FACSCanto II (BD Biosciences). The production of IFNγ and p21 was measured by intracellular staining and flow cytometry. BMMCs and PBMCs were stimulated for 4 h with 30 ng/ml PMA and 500 ng/ml ionomycin in the presence of 10 mg/ml BFA. After the surface staining cells were fixed and permeabilised using the Cytofix/Cytoperm kit (BD Pharmingen), and incubated with intracellular antibodies. Cells were washed and measured using a FACSCanto II (BD Biosciences). Detailed information on the antibodies used can be found in Additional file 1: Table S1. Dead cells were excluded using a fixable viability dye (Zombie Violet™ Fixable Viability Kit, Biolegend). Flow cytometry data were analysed using FlowJo v10 software.

### Antibody concentration measurement

Diphtheria-specific antibodies were measured in plasma obtained from peripheral blood. Microtiter plates were coated with 1 μg/ml diphtheria toxoid (Statens Serum Institute) and blocked with 0.01 M Glycin. Peroxidase-labelled rabbit anti-human IgG antibody (Chemicon/Millipore) was used as secondary antibody. Specific IgG antibodies were quantified in IU/ml using standard human anti-diphtheria sera (National Institute for Biological Standards and Control). The detection limit was 0.01 IU/ml, and values below this concentration were set to 0.005 IU/ml for calculation of geometric mean concentrations (GMC). Ab concentrations above 0.1 IU/ml were considered protective [28].

### Isolation of RNA and quantitative RT-PCR

RNA was isolated from purified BMMCs using the RNeasy Plus mini kit (Qiagen). First-strand cDNA synthesis was done using a reverse transcription system (Promega). Quantitative RT-PCR experiments were done using the LightCycler 480 System (Roche Diagnostics), 2× SYBR Green 1 Master (Roche Diagnostics), and β-actin as housekeeping gene for relative quantification of effector/memory cell survival factors. Sequence-specific oligonucleotide primers were designed using Primer3 software [26] and synthesized by MWG Biotech (Ebersberg, Germany). The following primers were used: IFNγFW 5′- GTAGCAATTGCCTGAATAATG-3′, IFNγRW 5′- GTTGTGCCTTCTGAAACT-3′, IL-15FW 5′-ATTTTGGGCTGTTTCAGTGC-3′, IL-15RW 5′-TTACTTTGCAACTGGGGTGA-3′; βACTINFW 5′-TCCTCCCTGGGCATGGAGT-3′, βACTINRW 5′-TCTCCTTCTGCATCCTGTCG-3′.

### Measurement of ROS

BMMCs and PBMCs were incubated with the fluorescent dye dihydroethidium (Sigma-Aldrich) at a concentration of 1:250 in complete RPMI for 20 min at 37 °C. Cells were washed in PBS, measured with a FACSCanto II (BD Biosciences).

### Statistical analysis

**Fig. 1:**
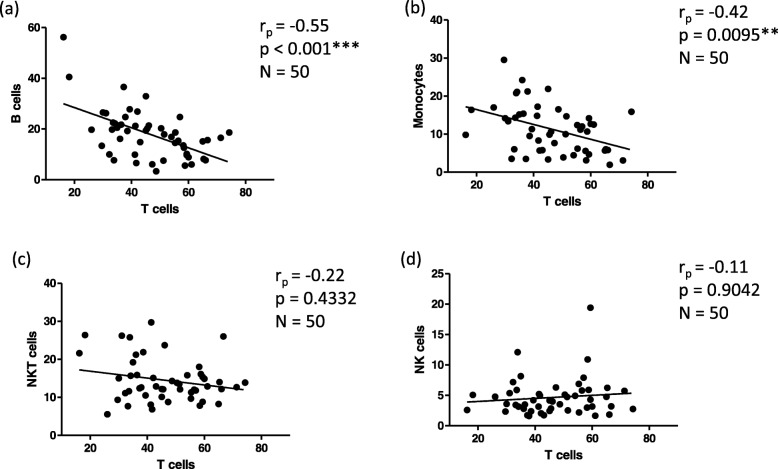
Correlations of T cells with B cells (**a**), Monocytes (**b**), NKT cells (**c**), and NK cells (**d**) in the human BM. T cells are defined as CD3^+^ lymphocytes (CD45^+^), B cells as CD19^+^ lymphocytes. Monocytes are defined as CD3^−^CD14^+^ lymphocytes, NKT cells as CD3^+^CD56^+^ lymphocytes, and NK cells as CD3^−^CD56^+^CD14^−^ lymphocytes. Correlation coefficients (r_p_) and significances were calculated according to Pearson with correction for age, values below 0.05 were considered significant; *p* < 0.05 is indicated by *, *p* < 0.01 is indicated by **, *p* < 0.001 is indicated by ***. N: number of samples

**Fig. 2:**
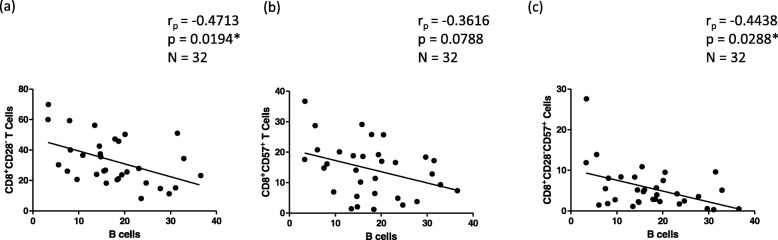
Correlations of B cells with highly-differentiated CD8^+^CD28^−^ T cells (**a**), immunosenescent/replicative senescent CD8^+^CD57^+^ (**b**), and highly-differentiated, replicative senescent CD8^+^CD28^−^CD57^+^ T cells in the human BM. B cells are defined as CD19^+^ lymphocytes, T cells are defined as CD3^+^ lymphocytes. Correlation coefficients (r_p_) and significances were calculated according to Pearson with correction for age

Pearson correlations were used to determine the statistical significance as indicated in the figure legends. *p* values less than 0.05 were considered significant. To exclude the influence of age on the correlations, partial correlations controlling for the variable age were performed using SPSS. With this method, the effect of age on the correlations has been removed completely. The correlation values calculated without controlling for age are shown in Additional file 1: Table S1. For the comparisons between populations (Figs. 1, 2 and Additional file 1: Table S1), p values were adjusted for multiple comparisons using Bonferroni corrections.

### Study approval

Study approval was given by the local institution, and written informed consent was received from all participants prior to their inclusion in the study in accordance with the Declaration of Helsinki.

## Results

### Competition between T cells and B cells in the BM

Major lymphocyte populations and sub-populations based on differentiation were analysed in BMMCs and PBMCs of 95 donors with an age range of 39 to 87 years. As the frequency of many populations, such as T cells, NKT cells, monocytes and B cells, increase in the BM with age (Table 1), all further calculations were statistically corrected for age. Correlation coefficients (r_p_) obtained considering the influence of age are reported in Additional file 1: Table S2. Gating strategy used to define these populations is shown in Additional file 2: Figure S1. Representative flow plots in young (31 years) and old (89 years) donors are shown in Additional file 2: Figure S2. Within the BM environment, strong negative correlations could be seen between T cells and B cells (*p* < 0.001) (Fig. 1a), and T cells and monocytes (*p* = 0.0095) (Fig. 1b). No correlation was found between T cells and NKT cells (Fig. 1c), or T cells and NK cells (Fig. 1d). These data suggest that populations in the BM may influence one another and that there may be a certain level of competition between T cells and B cells, as well as monocytes.

**Table 1:** Correlations of cell populations in the human BM and PB with age

	BM			PB		
	r_p_	p	N	r_p_	p	N
Total T cells	0.393	0.005	50	0.098	0.500	50
CD4 T cells	0.302	0.033	50	−0.003	0.983	50
CD8 T cells	−0.265	0.063	50	0.120	0.406	50
NKT cells	−0.305	0.031	50	0.152	0.291	50
NK cells	0.27	0.058	50	−0.094	0.517	50
Monocytes	−0.299	0.035	50	−0.144	0.317	50
Classical Monocytes	0.19	0.186	50	−0.232	0.105	50
Non-classical monocytes	−0.205	0.153	50	0.251	0.079	50
CD19^+^ cells	−0.316	0.026	50	−0.337	0.017	50
Plasma cells	0.195	0.175	50			
CD4^+^CD57^+^ T cells	−0.161	0.265	50	0.251	0.079	50
CD8 T cell populations						
Naïve	−0.319	0.024	50	−0.271	0.057	50
Central memory	−0.009	0.950	50	−0.273	0.055	50
Effector memory	−0.173	0.230	50	−0.090	0.535	50
Temra	0.369	0.008	50	0.412	0.003	50
CD8^+^CD28^−^ T cells	0.061	0.680	50	0.343	0.015	50
CD8^+^CD57^+^ T cells	0.265	0.258	50	0.385	0.006	50
CD8^+^CD28^−^CD57^+^ T cells	0.063	0.071	50	0.377	0.007	50

Correlation coefficients (r_p_) and significances were calculated according to Pearson with correction for age, values below 0.05 were considered significant. N: number of samples. T cells are defined as CD3^+^ Lymphocytes (CD45^+^), NKT cells as CD3^+^CD56^+^ Lymphocytes, NK cells as CD3^−^CD56^+^CD14^−^ Lymphocytes, Monocytes are defined as CD3^−^CD14^+^ lymphocytes, and B cells as CD19^+^ Lymphocytes

### Highly differentiated CD8^+^ T cells negatively influence B cells in the BM

The surface markers CD28 and CD57 were used to define populations of highly differentiated CD8^+^ T cells (Additional file 2: Figure S1). To determine if the accumulation of these highly differentiated CD8^+^ T cell subsets may negatively affect the maintenance of other cell populations we correlated the frequency of CD28^−^, CD57^+^, and CD28^−^CD57^+^CD8^+^ T cells with B cell frequency in the BM.

Interestingly, B cells correlated negatively with the levels of CD8^+^CD28^−^ (*p* = 0.0194) (Fig. 2a), CD8^+^CD57^+^ (*p* = 0.0788) (Fig. 2b), and CD8^+^CD28^−^CD57^+^ (*p* = 0.0288) T cells (Fig. 2c). These data suggest that B cells in the BM may be influenced by highly differentiated/terminally differentiated CD8^+^ T cells.

### Pro-inflammatory molecules are negatively associated with B cell frequency in the BM

**Fig. 3:**
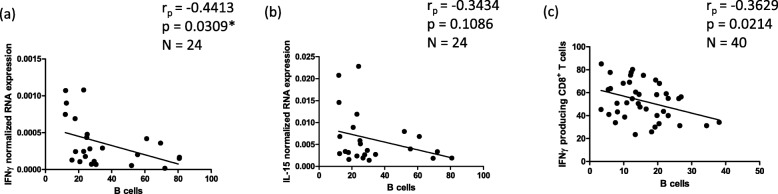
Correlations of B cells with the normalized RNA expression of both IFNγ (**a**) and IL-15 (**b**), and IFNγ producing CD8^+^ T cells (**c**) in the human BM. B cells are defined as CD19^+^ lymphocytes, T cells are defined as CD3^+^ lymphocytes. Cells were stimulated with PMA and Ionomycin for 4 h at 37 °C. Correlation coefficients (r_p_) and significances were calculated according to Pearson with correction for age

An elevated baseline level of pro-inflammatory markers, known as “inflammaging”, occurs during aging, therefore we quantified the expression of IFNγ and IL-15 in BMMCs at the mRNA level. IL-15 is produced by some BM cells and postulated to support highly differentiated, and therefore more pro-inflammatory T cells [7] .The expression levels of IFNγ negatively correlated with B cells in the BM (*p* = 0.03) (Fig. 3a), while a negative trend could be seen between IL-15 expression levels and B cells in the BM (*p* = 0.10) (Fig. 3b). In addition, the frequency of T cells producing IFNγ after stimulation negatively correlated with the percentage of B cells in the BM (p = 0.02) (Fig. 3c). From these data we conclude that, in addition to highly differentiated T cells, a pro-inflammatory environment may also have a negative impact on B cell maintenance in the BM.

### Concentrations of diphtheria-specific antibodies in the plasma correlate with cell populations, cellular senescence and ROS in the BM

**Fig. 4:**
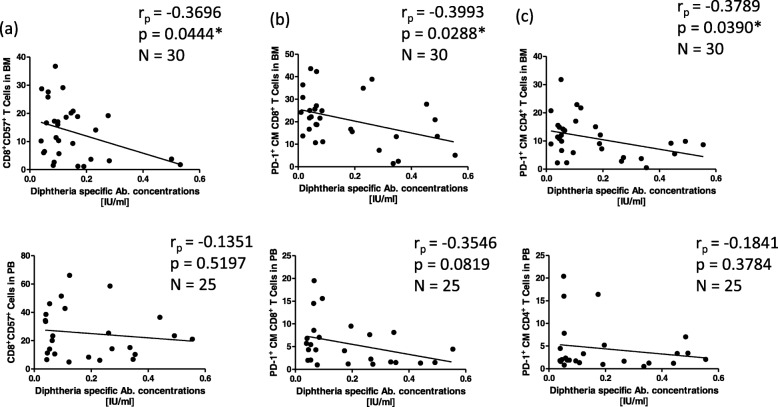
Diphtheria-specific antibody concentrations correlated with CD8^+^CD57^+^ T cells (**a**), CM CD8^+^ T cells expressing PD-1 (**b**), and CM CD4^+^ T cells expressing PD-1 (c) in the BM (upper panels), and in the PB (lower panels). CM: central memory T cells (CCR7^+^CD45RA^−^). Correlation coefficients (r_p_) and significances were calculated according to Pearson with correction for age

The support of long-lived plasma cell survival in the BM is thought to be mediated by cells in the BM niches [26]. Thus we hypothesized that changes in the BM environment affect antibody concentrations in the periphery. Diphtheria-specific antibody concentrations were measured in the plasma and correlated with cell populations from the BM and PB to indicate possible links between antibody concentrations in the blood and the BM environment. We investigated markers of differentiation, such as CD57, markers of exhaustion, such as PD-1, markers of cellular senescence, including p21 and KLRG-1, and the presence of ROS as an indicator for oxidative stress. Gating strategy used to define these populations is reported in Additional file 2: Figures S3-S4. Negative correlations were observed between diphtheria-specific antibody concentrations and highly differentiated CD8^+^CD57^+^ T cells in the BM (*p* = 0.044), whereas no correlation could be seen with cells in the PB (Fig. 4a). PD-1^+^ CM CD8^+^ T cells and PD-1^+^ CM CD4^+^ T cells in the BM (*p* = 0.029 & 0.039 respectively) (Fig. 4b and c – upper panels), negatively correlated with peripheral antibody concentrations. PD-1^+^ CM CD8^+^ T cells derived from peripheral blood showed a similar trend, but this correlation was not statistically significant (Fig. 4b and c - lower panels). These data support the hypothesis that the accumulation of senescent and/or exhausted T cells in the BM, negatively affect peripheral antibody concentrations.

**Fig. 5:**
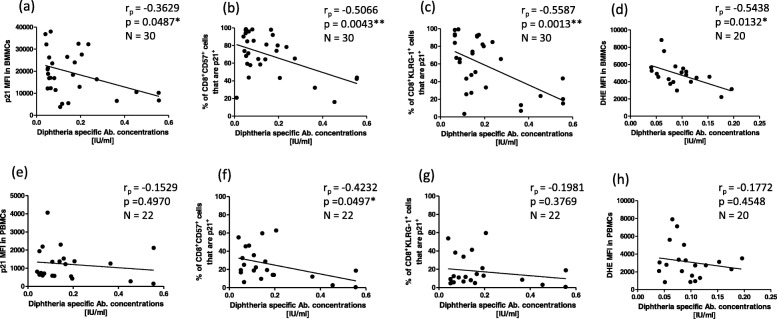
Diphtheria-specific antibody concentrations were correlated with; the mean fluorescence intensity of p21 (**a**), CD8^+^CD57^+^ T cells expressing p21 (**b**), CD8^+^ T cells expressing KLRG-1 and p21 (**c**), and Reactive oxygen species (**d**): in the BM or all BMMCs (upper panels) and in the PB or all PBMCs (lower panels). Cells were stimulated with PMA and Ionomycin for 4 h at 37 °C. Correlation coefficients (r_p_) and significances were calculated according to Pearson with correction for age

The cellular senescence marker p21 was measured in total BMMCs as well as in different T cell sub-populations (Fig. 5). Diphtheria-specific antibody concentrations negatively correlated with the mean fluorescence intensity (MFI) of p21 in all BMMCs (*p* = 0.0487), as well as with the percentage of CD8^+^CD57^+^and CD8^+^KLRG-1^+^ BM T cells expressing p21 (*p* = 0.0043 & 0.0013 respectively) (Fig. 5a-c, upper panels). In contrast, in the PB, this correlation was only observed for CD8^+^CD57^+^ T cells (*p* = 0.0497), when the same populations were analysed (Fig. 5a-c, lower panels). The levels of ROS were additionally measured, and a strong negative correlation could be seen between diphtheria-specific antibody concentrations and ROS levels in BMMCs (*p* = 0.0132) (Fig. 5d, upper panel), but not with ROS levels in PBMCs (Fig. 5d, lower panel). These data further corroborate our findings that senescent cells and/or highly-differentiated cells, as well as elevated ROS levels in the BM can negatively impact peripheral antibody concentrations.

## Discussion

The composition of immune cells in the BM changes with aging, and a shift from a naïve, to highly differentiated populations can be observed [29]. This shift is often used to describe immunosenescence [29]. As chronic infection, or repeated T cell activation can also drive these changes within the T cell compartment, Cytomegalovirus (CMV), is often referred to as a contributor to immunosenescence in the PB [30] and the BM [11]. CMV, a lifelong-persisting virus from the herpes virus family present in 60–100% of the elderly population (depending on the cohort), causes irreversible changes within the CD8^+^ T cell compartment, and the T cell repertoire of a young CMV–seropositive individual is often somewhat similar to an old CMV-seronegative repertoire. [11, 31]. The changes which we observed were even more pronounced in CMV positive individuals (data not shown).

The BM is important for the maintenance of antigen-experienced adaptive immune cells, in particular long-lived immune cells which populate survival niches in the BM [32]. After antigenic stimulation, effector/memory T cells and long-lived plasma cells accumulate within BM survival niches where they can be maintained for an undefined amount of time [33]. Our lab has previously shown that highly differentiated CD8^+^ T cells accumulate in the BM [7, 9]. The phenotype of these effector cells may be affected by the BM environment, or the different cell populations simultaneously present in the BM may interact and compete for space and/or the possibly limited survival factors available in the BM.

Among the immunological changes of the BM seen during aging, the percentage of T cells increases, in contrast to the PB where both the number and functionality decrease [34]. We questioned if the increase in T cell numbers in the BM could be due to the accumulation of senescent or exhausted cells T cells, thus affecting other cell populations, in particular B cells, in the BM. The negative correlations between both T cells and B cells, and T cells and monocytes independent of age, incite the idea that these events could be related.

IL-7 is a key survival factor for memory CD4^+^ and CD8^+^ T cells, acting as a central regulator for their survival and homeostasis [35]. In addition to this, IL-7 represents an important B cell factor, supporting B cell development, and regulating the proliferation and survival of B cell progenitors [36]. Other studies have shown that IL-7 supports B cells indirectly by inducing CD70 and BAFF expression in resting memory T cells, which in turn stimulate memory B cell activation and antibody production [37]. This cytokine has also been shown to play an important role in regulation of monocytes/macrophages [38]. Thus, as different immune cell populations share the same survival factor IL-7, which is produced by stromal cells located in restricted areas within the marrow [14], we can hypothesized that IL-7 may play an important role in the competition for space, at least between T cells, B cells and monocytes. Indeed, negative correlations between B and T cells, and monocytes and T cells in the BM have been observed in our study.

With age, more and more T cells lose the surface co-stimulatory molecule CD28, which is important for T cell activation [39]. Several causes are attributed to the loss of CD28 on the surface of T cells including chronic antigen stimulation and repeated T cell activation [10]. In addition to the loss of CD28, some cells gain expression of CD57, which is associated with an inability to proliferate, as well as high cytotoxic potential [40]. Therefore these CD8^+^CD28^−^CD57^+^ T cells are considered terminally differentiated T cells [10]. The strong negative correlations between B cells and CD8^+^CD57^+^, CD8^+^CD28^−^, and CD8^+^CD28^−^CD57^+^ T cells, suggest that these highly differentiated T cells affect B cell maintenance in the BM. In contrast to T cells of earlier differentiation stages, highly differentiated T cells are less responsive to IL-7, as they express lower levels of IL-7Rα [41]. It therefore seems unlikely that they compete with B cells for this cytokine [41], but rather that the correlations are a result of an indirect effect. The accumulation of highly differentiated T cells, leading to increased levels of pro-inflammatory cytokines and ROS [11] might additionally result in a stressful environment for B cells [42].

With age, a quantitative and qualitative decline of B cell responses can be observed. It has previously been pointed out, that functional alterations in aged T cells contribute to the defects in B cell function [43]. CD19^+^ B cells are known to decrease in the BM with age [43], and the expression of the adhesion molecules CD49d and CD50, which are important for B cell adhesion to epithelium, are reduced in elderly subjects [44]. IFNγ in particular, produced in higher amounts by terminally differentiated T cells, has been described to inhibit B cell differentiation [45]. The quantitative and qualitative decline of B cell responses, and disadvantageous intrinsic changes in B cells have also previously been explored, specifically independent of T cell influence, as defective aged T cells are suspected to contribute to B cell decline [43]. We first considered the mRNA expression of IFNγ and IL-15 in all BMMCs, and we saw a significant negative correlation between IFNγ and B cells in the BM. In addition to the mRNA expression we also considered protein expression of IFNγ by individual cells and found strong negative correlations between B cells and pro-inflammatory IFNγ producing CD8^+^ T cells. We also found strong negative correlations between B cells and pro-inflammatory IFNγ producing CD8^+^CD57^+^, CD8^+^CD28^−^, and CD8^+^CD28^−^CD57^+^ T cells (data not shown), highlighting the negative influence that a pro-inflammatory environment can have on B cells. Highly differentiated T cells which accumulate in the BM are more pro-inflammatory [11], and a pro-inflammatory environment can negatively impact B cell development [45]. After B cells are activated, they rapidly proliferate and undergo somatic hyper mutation, changing the affinity of their Ig variable regions [46]. These “class-switched” B cells are experienced immune cells and have been reported to be higher in the peripheral blood of smokers [47]. All of this considered, suggests that the loss of B cell diversity is strongly associated with poor health rather than age [48], and that inflammation clearly has a large effect on BM cell populations. The presence of IFNγ in the bone due to antigen-driven T cell activation has previously been shown to stimulate osteoclast formation, resulting in bone loss [49], further highlighting the dramatic effects that inflammation can have on the bone/BM. These data support out hypothesis that highly differentiated CD8^+^ T cells accumulating in the BM not only support inflammation, but also directly affect B cell maintenance. Peripheral antibody concentrations are heavily dependent on long-living, antibody-producing, plasma cells in the BM [50]. This is clinically relevant as serum antibodies ensure protection after vaccination and in case of repeated exposure to the same pathogen [51]. We considered the possibility that the BM environment, and the accumulation of highly differentiated T cells may affect peripheral antibody concentrations. We investigated diphtheria-specific antibody concentrations as they are induced by one of the most commonly applied vaccines world-wide and have been demonstrated to be poorly maintained in the elderly [27].

Negative correlations were seen between diphtheria-specific antibody concentrations and highly differentiated CD8^+^CD57^+^ T cells, exhausted PD-1^+^ CM CD8^+^ T cells, and PD-1^+^ CM CD4^+^ T cells in the BM. PD-1 can be expressed on activated T cells, and does not always signify an exhausted cell. PD-1 expression was found to be inversely correlated with the expression of CD45RA, with memory cells expressing the highest proportions of PD-1 [52]. We used the PD-1^+^ CM cell populations to represent an exhausted population because CCR7, a homing marker for lymphoid tissue which is expressed on CM T cells, was almost undetectable in PD-1 expressing CD8^+^ T cells [24], and a high expression of PD-1 has been observed on EM CD8^+^ T cells in the PB of healthy humans [52]. Cellular senescence in the BM, indicated by expression of p21 in total BMMCs, as well as in highly-differentiated and/or senescent CD8^+^CD57^+^ and senescent CD8^+^KLRG-1^+^ T cells was associated with lower diphtheria-specific antibodies in the periphery. Elevated ROS levels also correlated with low antibody concentrations. The corresponding T cell populations in the peripheral blood barely influenced antibody concentrations. Unfortunately, no information about diphtheria vaccination was available for our samples. As our cohort includes a mixture of recently vaccinated individuals and persons vaccinated several years before, these aspects compensate each other in the correlations. Despite this, in our study it is not possible to discriminate between recently vaccinated donors with low Ab concentrations and donors vaccinated many years before.

In summary, these results show that cellular senescence, ROS, and the accumulation of senescent CD8^+^ T cells in the BM, but not in the periphery, can alter antibody production by long-lived plasma cells in the BM, leading to reduced antibody concentrations.

## Conclusions

Our work provides further evidence of the important role the BM plays in regulating the survival of memory and effector cells. Changes in the BM environment, or accumulation of certain populations may affect the survival of “healthy” memory cells and plasma cells, leading to impaired antibody production. A better understanding of these effects may help us in developing more successful approaches to maintaining life-long protective antibody titres. Strategies to fight against cellular senescence, ROS and inflammation in the BM should be addressed in future studies in order to guarantee a working adaptive immunity in the elderly.

## Additional files

Additional file1: **Table S1.** Antibodies used in surface and intracellular staining for flow cytometry. **Table S2.** Comparison between correlations corrected or not corrected for age. Correlation coefficients (rp) and significances were calculated according to Pearson with correction for age, values below 0.05 were considered significant. N: number of samples. (DOCX 45 kb)

Additional file 2: **Figure S1.** Gating strategy for the populations of interest in the BM shown in Figures 1-3. **Figure S2.** Representative flow plots showing the frequency of (CD3+) T cells (a), (CD19+) B cells (b) and CD14+ monocytes (c) in a young (31 years) and an old (89 years) donors. **Figure S3.** Gating strategy for the populations of interest in the BM shown in Figures 5-6. **Figure S4.** Gating strategy for the populations of interest in the PB shown in Figures 4-5. (PPTX 255 kb)

## Data Availability

The datasets used and/or analysed during the current study are available from the corresponding author on reasonable request.

## Abbreviations

BM: bone marrow
BMMCs: BM mononuclear cells
NKT: natural killer T cells
PB: peripheral blood
PBMCs: PB mononuclear cells
ROS: reactive oxygen species
T_EM_: effector memory T cells
T_EMRA_: effector memory cells re-expressing CD45RA
