# Supplementary material for: Peripheral antibody concentrations are associated with highly differentiated T cells and inflammatory processes in the human bone marrow
Source: Immun Ageing. 2019 Aug 22;16:21. doi: 10.1186/s12979-019-0161-z (PMC6706884; doi:10.1186/s12979-019-0161-z)
Supplement: Supplementary file 1 — Table S1. Antibodies used in surface and intracellular staining for flow cytometry. Table S2. Comparison between correlations corrected or not corrected for age. Correlation coefficients (rp) and significances were calculated according to Pearson with correction for age, values below 0.05 were considered significant. N: number of samples. (DOCX 45 kb) [file 12979_2019_161_MOESM1_ESM.docx]

**Supplementary table 1**: Antibodies used in surface and intracellular staining for flow cytometry

| **Antibody** | **Clone** | **Fluorochrome** | **Company** |
| --- | --- | --- | --- |
| **Surface Antibodies** |  |  |  |
| α-CD3 | REA613 | VioGreen | Miltenyi |
| α-CD3 | REA613 | APC-Vio | Miltenyi |
| α-CD4 | REA623 | VioGreen | Miltenyi |
| α-CD4 | REA623 | APC | Miltenyi |
| α-CD8 | REA734 | PE-Cy7 | Miltenyi |
| α-CD57 | TB03 | FITC | Miltenyi |
| α-CCR7 | REA546 | FITC | Miltenyi |
| α-CD138 | 44F9 | APC | Miltenyi |
| 7-AAD | Live/Dead | PerCP | Miltenyi |
|  |  |  |  |
| α-CD16 | 3G8 | FITC | BD |
| α-CD56 | NCAM16.2 | PE | BD |
| α-CD28 | CD28.2 | BV421 | BD |
| α-CD25 | M-A251 | V500 | BD |
| α-CD3 | UCHT1 | FITC | BD |
| α-CD69 | FN50 | PE | BD |
| α-CD27 | M-T271 | BV421 | BD |
| α-CD45 | HI30 | V500 | BD |
| α-IgD | IA6-2 | FITC | BD |
| α-CD19 | HIB19 | PE | BD |
| α-CD20 | 2H7 | PE-Cy7 | BD |
| α-CD38 | HB-7 | APC | BD |
| α-CD38 | HIT2 | V500 | BD |
| α-CD3 | UCHT1 | PE | BD |
| α-CD19 | SJ25C1 | APC-Cy7 | BD |
|  |  |  |  |
| α-CD14 | HCD14 | PE-Cy7 | Biolegend |
| α-CD45 | SK7 | (APC-H7)-APC-eFluor 780 | Biolegend |
| α-CD57 | HNK-1 | PE | Biolegend |
| α-CD45 | UCHL1 | APC | Biolegend |
| α-PD-1 | EH12.2H7 | PE | Biolegend |
| α-CD8 | SK1 | PerCP | Biolegend |
| α-KLRG-1 | 2F1/KLRG1 | PE-Cy7 | Biolegend |
|  |  |  |  |
| α-IL-7Rα | eBioRDR5 | APC | eBioscience |
|  |  |  |  |
| **Intracellular Antibodies** | |  |  |
| α-CD8 | REA734 | VioGreen | Miltenyi |
| α-CD28 | REA612 | PE-Vio770 | Miltenyi |
| α-CD3 | REA613 | APC-Vio | Miltenyi |
| α-KLRG-1 | REA261 | PE-Vio770 | Miltenyi |
| α-γH2Ax | REA502 | FITC | Miltenyi |
|  |  |  |  |
| α-PD-1 | EH12.247 | PE | Biolegend |
| α-CD57 | HNK-1 | PerCP | Biolegend |
|  |  |  |  |
| α-CD28 | CD28.2 | BV421 | BD |
| α-TNFα | Mab11 | FITC | BD |
| α-IL-2 | MQ1-17H12 | PE | BD |
| α-IFNγ | B27 | APC | BD |
| α-p16 | Set | PE | BD |
|  |  |  |  |
| α-IL-7Rα | eBioRDR5 | APC | eBiosceince |
| α-IL-17 | eBio64DEC17 | PE | eBiosceince |
| α-IL-10 | BT-10 | FITC | eBiosceince |
|  |  |  |  |
| α-p21 | F5 | FITC | Santa Cruz |

|  | **age correction** | | **no age correction** | |
| --- | --- | --- | --- | --- |
| **T cells** | **r_p_** | **p** | **r_p_** | **p** |
| **vs B cells** | -0.55 | <0.001 | -0.15 | 0.5348 |
| **vs monocytes** | -0.42 | 0.0095 | -0.44 | 0.0032 |
| **vs NKT cells** | -0.22 | 0.4332 | -0.21 | 0.4452 |
| **vs NK cells** | -0.11 | 0.4437 | 0.05 | 0.7521 |
|  |  |  |  |  |
| **B cells** | **r_p_** | **p** | **r_p_** | **p** |
| **vs CD8^+^CD28^-^ T cells** | -0.47 | 0.0194 | -0.52 | 0.012 |
| **vs CD8^+^CD57^+^ T cells** | -0.36 | 0.0788 | -0.38 | 0.062 |
| **vs CD8^+^CD28^-^CD57^+^ T cells** | -0.44 | 0.0288 | -0.41 | 0.041 |
| **vs IFNγ mRNA** | -0.44 | 0.0309 | -0.51 | 0.05 |
| **vs IL-15 mRNA** | -0.34 | 0.1086 | -0.13 | 0.65 |
| **vs IFNγ^+^CD8^+^ T cells** | -0.36 | 0.0214 | -0.29 | 0.16 |
|  |  |  |  |  |
| **Diphteria Ab conc** | **r_p_** | **p** | **r_p_** | **p** |
| **vs CD8^+^CD57^+^ T cells** | -0.37 | 0.0444 | -0.31 | 0.136 |
| **vs CM CD8^+^ T cells** | -0.40 | 0.0288 | -0.29 | 0.152 |
| **vs PD-1^+^ CM CD4^+^ T cells** | -0.38 | 0.039 | -0.37 | 0.0179 |
| **vs p21 MFI in BMMCs** | -0.36 | 0.0487 | -0.32 | 0.0621 |
| **vs p21^+^CD8^+^CD57^+^ T cells** | -0.51 | 0.0043 | -0.47 | 0.021 |
| **vs p21^+^KLRG-1^+^CD8^+^ T cells** | -0.56 | 0.0013 | -0.55 | 0.0022 |
| **vs ROS in BMMCs** | -0.54 | 0.0132 | -0.46 | 0.0301 |

**Supplementary table 2**: Comparison between correlations corrected or not corrected for age

Correlation coefficients (r_p_) and significances were calculated according to Pearson with correction

for age, values below 0.05 were considered significant. N: number of samples.
