# Supplementary material for: Peripheral antibody concentrations are associated with highly differentiated T cells and inflammatory processes in the human bone marrow
Source: Immun Ageing. 2019 Aug 22;16:21. doi: 10.1186/s12979-019-0161-z (PMC6706884; doi:10.1186/s12979-019-0161-z)
Supplement: Supplementary file 2 — Figure S1. Gating strategy for the populations of interest in the BM shown in Figures 1-3. Figure S2. Representative flow plots showing the frequency of (CD3+) T cells (a), (CD19+) B cells (b) and CD14+ monocytes (c) in a young (31 years) and an old (89 years) donors. Figure S3. Gating strategy for the populations of interest in the BM shown in Figures 5-6. Figure S4. Gating strategy for the populations of interest in the PB shown in Figures 4-5. (PPTX 255 kb) [file 12979_2019_161_MOESM2_ESM.pptx]

## Slide 1
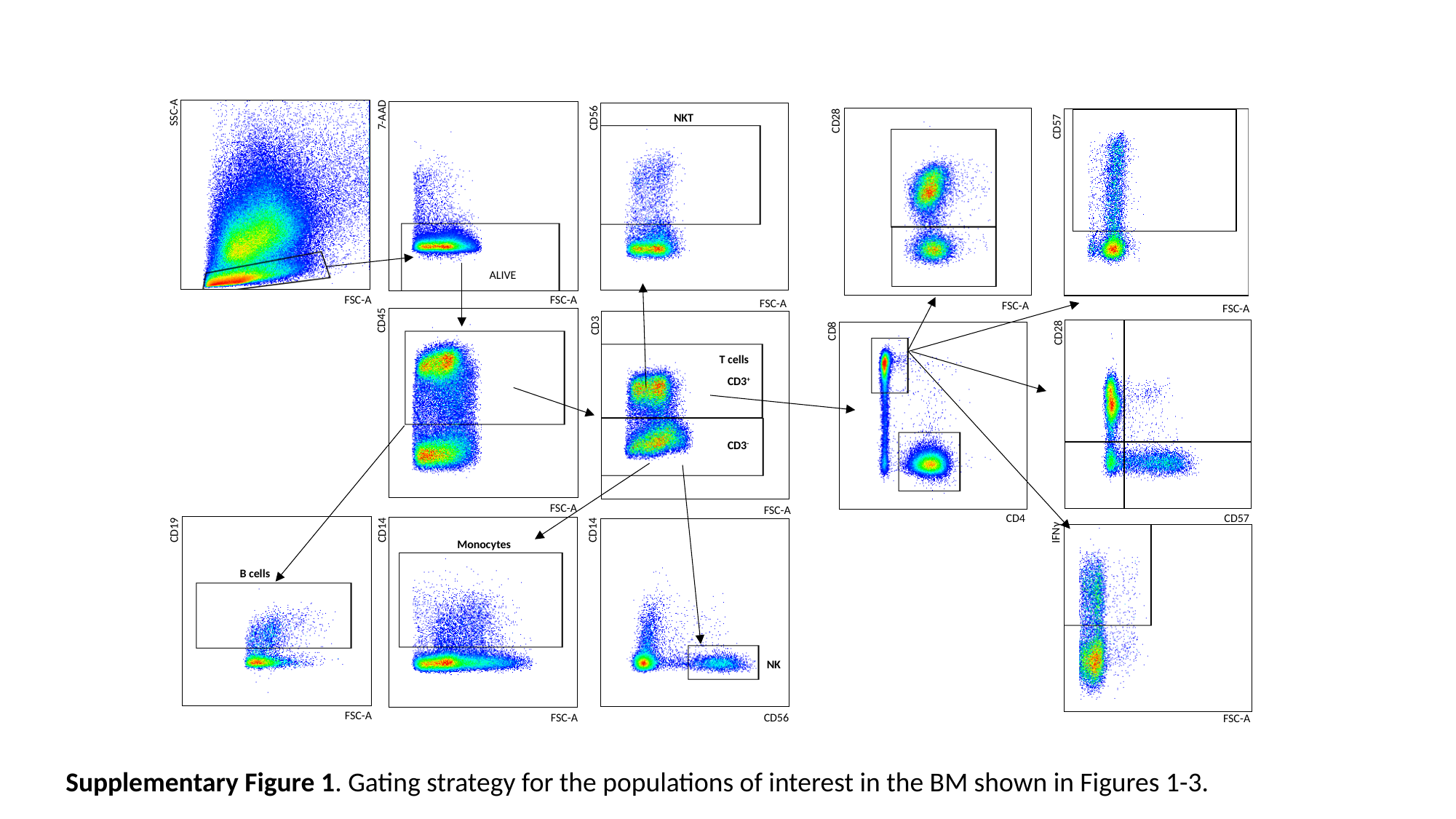

SSC-A
FSC-A
7-AAD
FSC-A
FSC-A
CD45
CD28
FSC-A
CD56
CD57
NKT
CD3
FSC-A
ALIVE
CD8
CD28
FSC-A
FSC-A
T cells
CD3+
CD19
FSC-A
CD14
FSC-A
CD3-
CD14
IFNγ
CD57
CD4
Monocytes
B cells
NK
CD56
FSC-A
Supplementary Figure 1. Gating strategy for the populations of interest in the BM shown in Figures 1-3.

## Slide 2
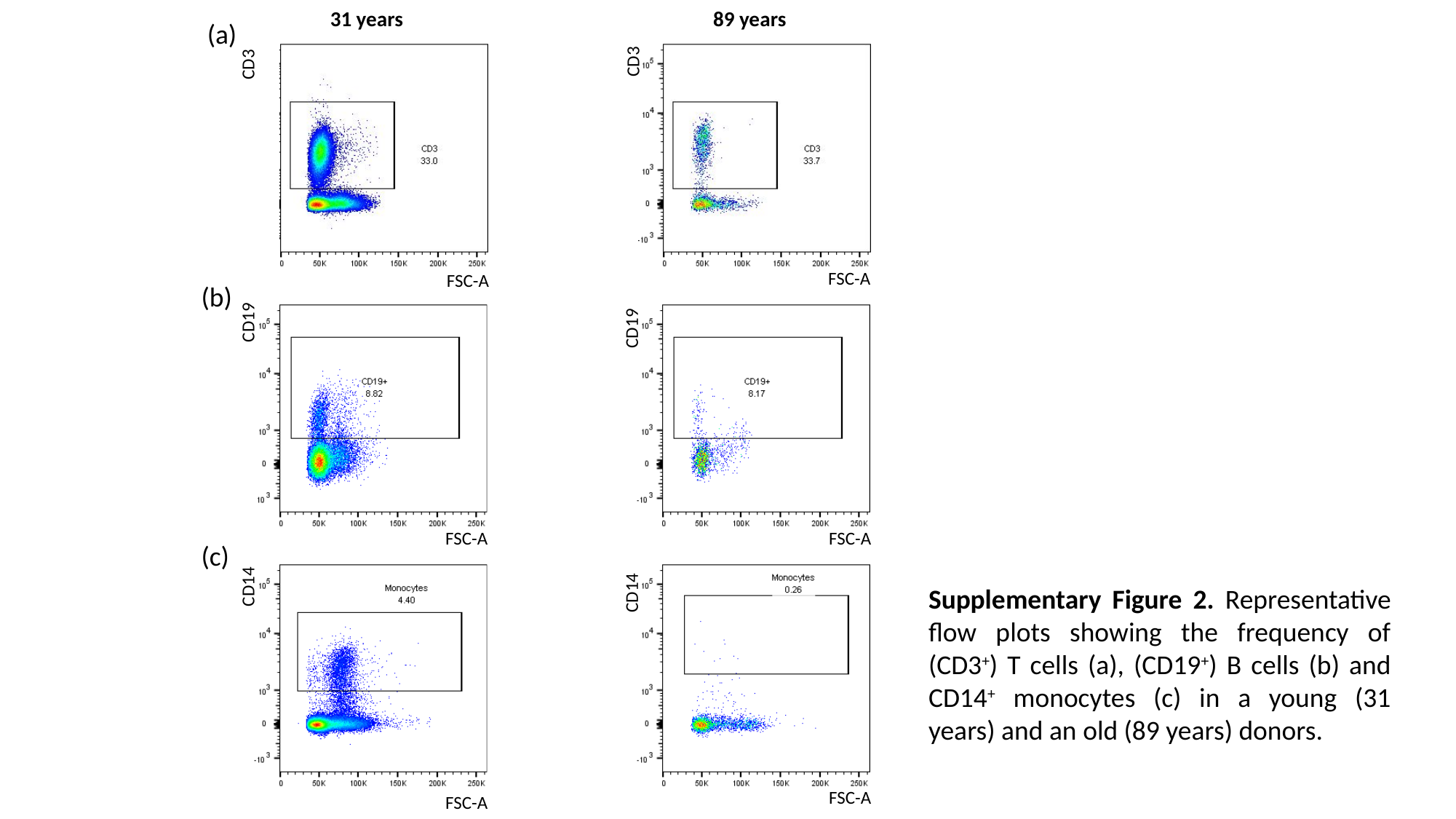

31 years
CD3
FSC-A
CD3
FSC-A
(a)
CD19
CD19
FSC-A
FSC-A
(b)
CD14
CD14
FSC-A
FSC-A
(c)
89 years
Supplementary Figure 2. Representative flow plots showing the frequency of (CD3+) T cells (a), (CD19+) B cells (b) and CD14+ monocytes (c) in a young (31 years) and an old (89 years) donors.

## Slide 3
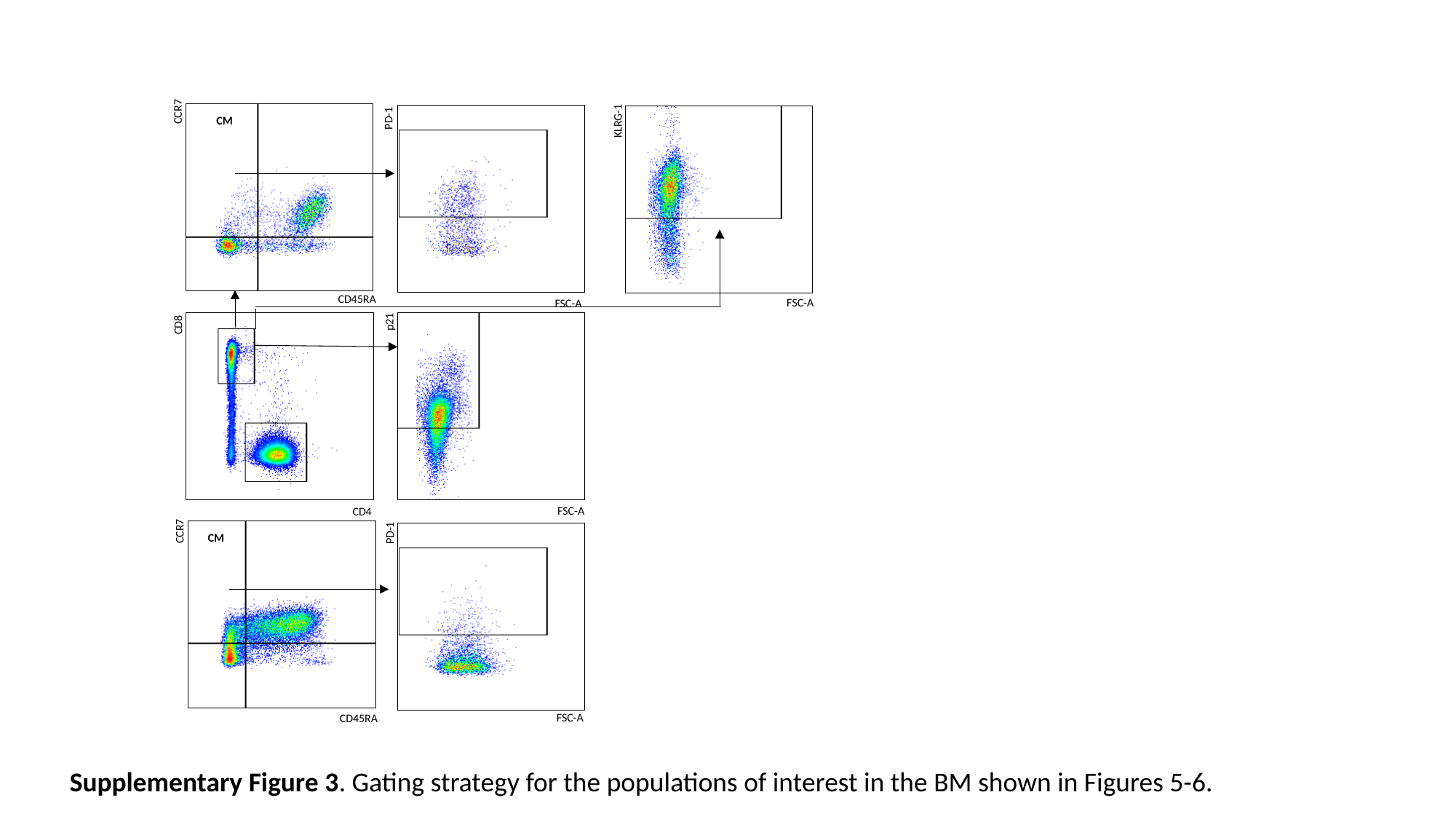

CCR7
PD-1
KLRG-1
CM
p21
CD8
CD45RA
FSC-A
FSC-A
CCR7
PD-1
FSC-A
CD4
CM
FSC-A
CD45RA
Supplementary Figure 3. Gating strategy for the populations of interest in the BM shown in Figures 5-6.

## Slide 4
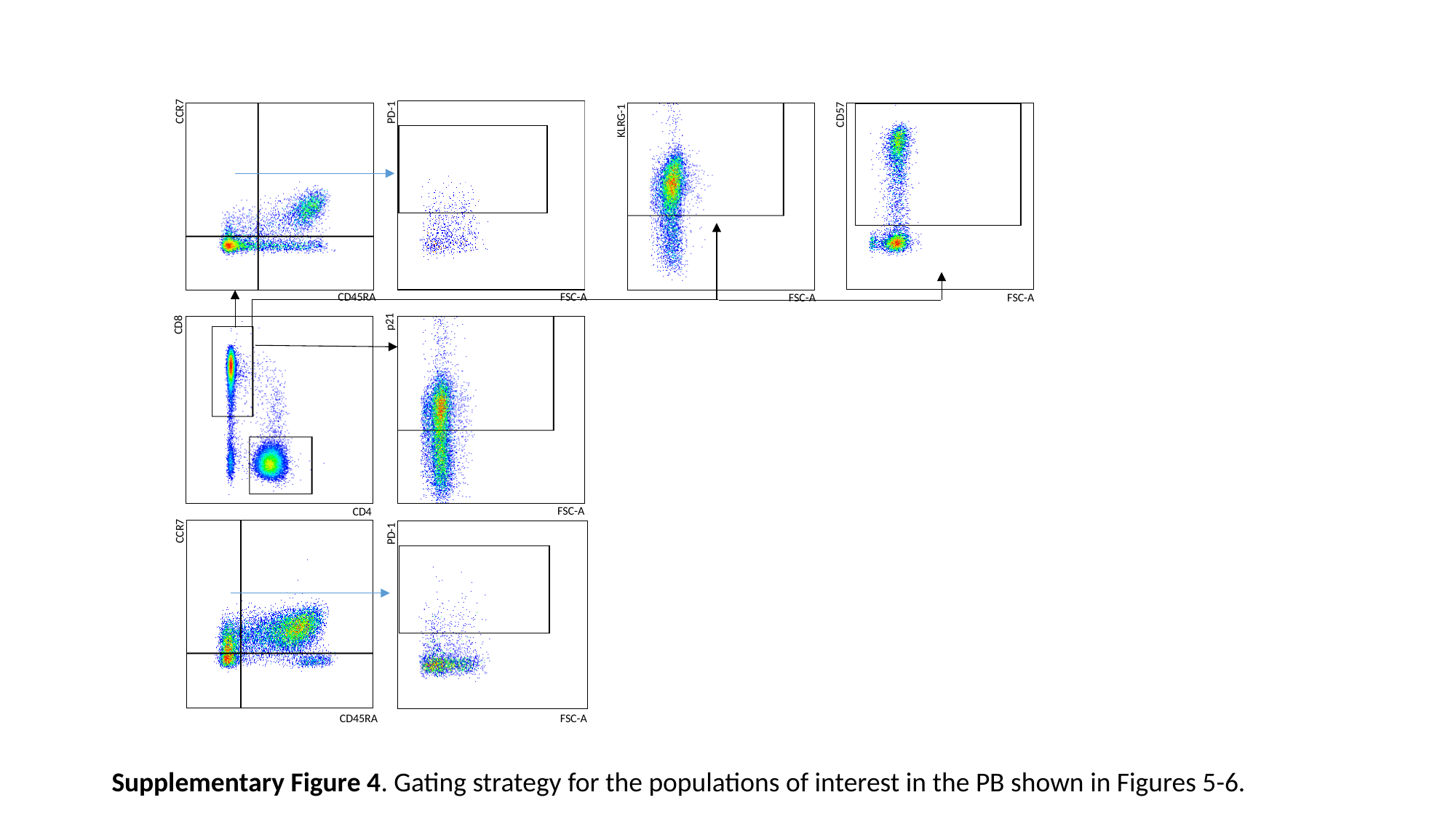

CCR7
PD-1
CD57
KLRG-1
p21
CD8
FSC-A
CD45RA
FSC-A
FSC-A
CCR7
PD-1
FSC-A
CD4
FSC-A
CD45RA
Supplementary Figure 4. Gating strategy for the populations of interest in the PB shown in Figures 5-6.
